# Consensus of Nonlinear Complex Systems with Edge Betweenness Centrality Measure under Time-Varying Sampled-Data Protocol

**DOI:** 10.1155/2015/493907

**Published:** 2015-06-18

**Authors:** M. J. Park, O. M. Kwon, E. J. Cha

**Affiliations:** ^1^School of Electrical Engineering, Chungbuk National University, 52 Naesudong-ro, Cheongju 361-763, Republic of Korea; ^2^Department of Biomedical Engineering, School of Medicine, Chungbuk National University, 52 Naesudong-ro, Cheongju 361-763, Republic of Korea

## Abstract

This paper proposes a new consensus criterion for nonlinear complex systems with edge betweenness centrality measure. By construction of a suitable Lyapunov-Krasovskii functional, the consensus criterion for such systems is established in terms of linear matrix inequalities (LMIs) which can be easily solved by various effective optimization algorithms. One numerical example is given to illustrate the effectiveness of the proposed methods.

## 1. Introduction

During the last few years, complex systems have received increasing attention from the real world such as the social networks, electrical power grids, global economic markets, small-world network, and scale-free network. Complex systems have the information flow which is consisted of a set of interconnected nodes with specific dynamics. For more details, see the literature [1–4] and the references therein. Also, many models has been proposed to describe multiagent systems, various coupled neural network, and so on [5–9].

Nowadays, most systems use microprocessor or microcontrollers, which are called digital computer. But the physical real situation is that the computers are on discrete signals while the plants are on continuous signals. In line with this thinking, in order to analyze the behavior of the plant between sampling instants, it is necessary to consider both the discrete operation of the computer and the continuous response of the plant. A little more to say, the fundamental character of the digital computer is that it takes the computed answers at sampling instants to calculate the control operation of a continuous plant. In addition to this, samples are taken from the continuous physical signals such as position, velocity, or temperature and these samples are used in the computer to calculate the controls to be applied. Systems in which discrete signals appear in some places and continuous signals occur in other parts are called sampled-data systems because continuous data are sampled before being used [10]. For this reason, various sampled-date control problems were investigated in [11–13]. Return to complex systems, this system is also booked for the consensus problem with sampled data [14–16].

However, there is room for further improvements in consensus analysis of complex systems. In most studies on complex systems such as multiagent system, complex dynamical network, and coupled neural network, the Laplacian matrix which is consisted of the adjacency and degree matrices of network is used. Because the foresaid matrices are based on degree centrality measure, the existing works need only the local structural information of network, that is, the degree centrality of node, which is determined by the number of nodes adjacent to it. Hence, by considering some other properties of graph theory, the structural information of network to analyze consensus problem for such system will be advanced. The edge betweenness centrality is selected from a choice among the properties of graph theory. Moreover, the edge betweenness centrality quantifies the average shortest path between two other nodes per each edge. It was introduced as a measure for quantifying the control of a human on the communication between other humans in a social network by [17, 18]. Thus, the edge between two nodes has strongly an impact on the overall structure of information flow. Sometimes, the nodes with small degree centrality are directly connected through edges with larger betweenness centrality [3]. In this case, such edges should be weighted by the value with proportional to their betweenness centrality. Therefore, through the edge betweenness centrality measure, not only the local structural information but also the global effects of structure of information flow are considered. As a result, the consensus analysis in complex systems will be advanced by weighting each edge to its betweenness centrality.

Motivated by what was mentioned above, in this paper, a consensus criterion for nonlinear complex systems with edge betweenness centrality measure under time-varying sampled-data protocol will be proposed in Theorem 6 with the frame work of LMIs [19]. For comparison, based on the results of Theorem 6, a consensus criterion for such system with degree centrality measure will be introduced in Corollary 7. Through one numerical example, it will be shown that the proposed model can give its usefulness.

**Notation 1 .**

*ℝ* ^*n*^ and *ℝ* ^*m*×*n*^ denote the *n*-dimensional Euclidean space with vector norm ‖·‖ and the set of all *m* × *n* real matrices, respectively. *𝕊* ^*n*^ and *𝕊*_+_^*n*^ are the sets of symmetric and positive definite *n* × *n* matrices, respectively. *I*_*n*_ denotes *n* × *n* identity matrix. *X* > 0 (<0) means symmetric positive (negative) definite matrix. *X* ^⊥^ stands for a basis for the nullspace of *X*. diag{⋯} represents the block diagonal matrix. For any square matrix *X* and any vectors *x*_*i*_, respectively, we define $sym\{X\}=X+X^{T}$ and $col\{x_{1},x_{2},\ldots ,x_{n}\}={\left[x_{1}^{T}\;x_{2}^{T}\cdots x_{n}^{T}\right]}^{T}$. The symmetric terms in symmetric matrices and in quadratic forms will be denoted by ⋆ (This is used if necessary.). *X*_[*f*(*t*)]_ means that the elements of matrix *X*_[*f*(*t*)]_ include the scalar value of *f*(*t*) affinely.

## 2. Problem Statements

Consider the model of nonlinear complex systems given by

(1) $$\begin{array}{c} {\dot{x}}_{i}\left(t\right)=Ax_{i}\left(t\right)+Bf\left(y_{i}\left(t\right)\right)+u_{i}\left(t\right),\\ y_{i}\left(t\right)=Cx_{i}\left(t\right),\;i=1,2,\ldots ,N, \end{array}$$

where *N* is the number of coupled nodes, *n* is the number of state of each node, the subscript *i* means the *i*th node, *x*_*i*_(*t*) ∈ *ℝ* ^*n*^ is the state vector, *y*_*i*_(*t*) ∈ *ℝ* ^*n*_*y*_^ is the output vector, *A* ∈ *ℝ* ^*n*×*n*^, *B* ∈ *ℝ* ^*n*×*n*_*y*_^, and *C* ∈ *ℝ* ^*n*_*y*_×*n*^ are system matrices, and *f*(·) ∈ *ℝ* ^*n*_*y*_^ denotes the nonlinearity, which satisfies *f*_*q*_(0) = 0 (*q* = 1,…, *n*_*y*_) and

(2) $$\begin{array}{c} l_{q}^{-}\leq \frac{f_{q}\left(u\right)-f_{q}\left(v\right)}{u-v}\leq l_{q}^{+},\;u\neq v,\;\;\forall u,v\in R, \end{array}$$

where *l*_*q*_^−^ and *l*_*q*_^+^ are given constants. For simplicity, let us define $L^{-}=diag\{l_{1}^{-},\ldots ,l_{n}^{-}\}$ and $L^{+}=diag\{l_{1}^{+},\ldots ,l_{n}^{+}\}$.

Let us consider the following consensus protocol proposed by [3]:

(3) uit=−σ∑j=1,j≠iNγij∑j=1,j≠iNγijxit−xjt,i=1,2,…,N,

where *σ* is a given scalar meaning the coupling strength, *γ*_*ij*_ is the edge betweenness centrality between nodes *i* and *j* defined by

(4) $$\begin{array}{c} \gamma_{ij}=\underset{k\neq l}{\sum }\frac{g_{kl}\left(e_{ij}\right)}{g_{kl}}, \end{array}$$

where $e_{ij}$ denotes the edge between nodes *i* and *j*, $g_{kl}$ is the number of the shortest paths from nodes *k* to *l* in the graph, and $g_{kl}(e_{ij})$ is the number of these shortest paths through path $e_{ij}$.

**Remark 1 .**

The consensus protocol (3) with edge betweenness centrality measure will be compared with the common consensus protocol followed by

(5) $$\begin{array}{c} u_{i}\left(t\right)=-\sigma \overset{N}{\underset{j=1,j\neq i}{\sum }}d_{ij}\left(x_{i}\left(t\right)-x_{j}\left(t\right)\right), \end{array}$$

where *d*_*ij*_ = 1 if node *i* is connected to node *j* and otherwise, *d*_*ij*_ = 0.

**Figure 1:**
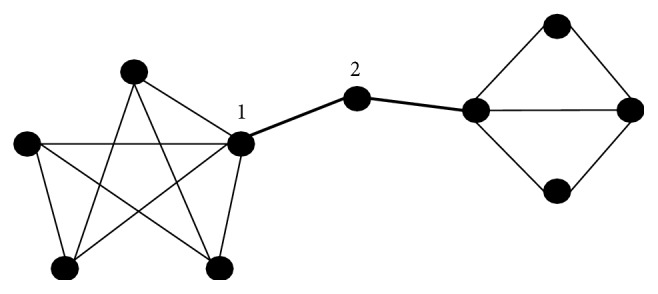
Structure example for information flow.

For details, from Figure 1, the thickness of edge is proportional to the edge betweenness centrality, which can be paraphrased as the load of edge. Thus, node 2 has the edge with the largest value of edge betweenness centrality compared to its smallest degree centrality while the degree centrality of node 1 is the largest value. As a guide, the degree centrality of node is determined by the number of nodes adjacent to it, for example, the value of node 1 is ∑_*j*≠*i*_ *d*_1*j*_ = 5, and in this sense, the common protocol (5) considers degree centrality measure. Therefore, in protocol (3), not only the local structural information but also the global effects of structure of information flow can be considered.

In this paper, the following protocol with the sampled-data information flow is proposed:

(6) uitk=−σ∑j=1,j≠iNγij∑j=1,j≠iNγijxitk−xjtk,i=1,2,…,N,

where *t*_*k*_ are the sampling instants satisfying 0 = *t*_0_ < *t*_1_ < ⋯<*t*_*k*_ < ⋯<lim_*k*→*∞*_ *t*_*k*_ = +*∞*. For its analysis, assume that the sampling interval is constant; that is, *t*_*k*+1_ − *t*_*k*_ = *h*_*M*_. Then, let us define

(7) $$\begin{array}{c} h\left(t\right)=t-t_{k},\;t\in \left[t_{k},t_{k+1}\right). \end{array}$$

Note that *h*(*t*) ≤ *h*_*M*_ and $\dot{h}(t)=1$ for *t* ≠ *t*_*k*_.

**Remark 2 .**

**Figure 2:**
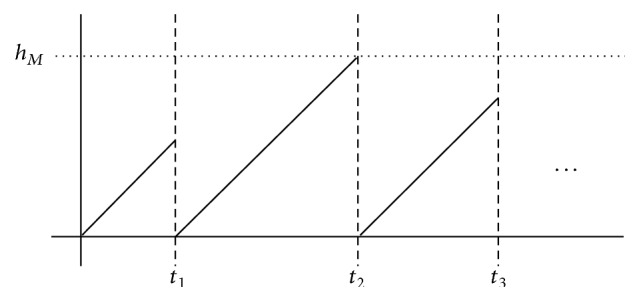
Time-varying sampling.

The consensus protocol (6) is assumed to be generated by using a zero-order-hold function with a sequence of hold times 0 = *t*_0_ < *t*_1_ < ⋯<*t*_*k*_ < ⋯. Then, the definition (7), *h*(*t*) = *t* − *t*_*k*_, is that the interval between two sampling instants is less than a given bound, *h*_*M*_ = *t*_*k*+1_ − *t*_*k*_. Hence, (7) means the time-varying sampling drawn as shown in Figure 2. In addition to the figure, all slopes are 1.

The aim of this paper is to analyze the consensus of the complex systems (1) under the time-varying sampled-data protocol (6) given by

(8) x˙it=Axit+BfCxit−σ∑j=1,j≠iNγij ×∑j∈NiNγijxitk−xjtk, i=1,2,…,N.

This means that the protocol *u*_*i*_(*t*_*k*_) solves the consensus problem, if and only if the states of each node satisfy

(9) $$\begin{array}{c} \underset{t\to \infty }{lim}\left\|x_{i}\left(t\right)-x_{j}\left(t\right)\right\|=0,\;i,j=1,2,\ldots ,N. \end{array}$$

The following lemmas will be used to derive the main result.

**Lemma 3 (see [6]).**

Let *U* = [*u*_*ij*_]_*N*×*N*_, *P* ∈ *ℝ* ^*n*×*n*^, $x=col\{x_{1},x_{2},\ldots ,x_{n}\}$, and $y=col\{y_{1},y_{2},\ldots ,y_{n}\}$. If *U* = *U* ^*T*^ and each row sum of *U* is zero, then

(10) $$\begin{array}{c} x^{T}\left(U⊗P\right)y=-\underset{1\leq i<j\leq N}{\sum }u_{ij}{\left(x_{i}-x_{j}\right)}^{T}P\left(y_{i}-y_{j}\right). \end{array}$$

**Lemma 4 (see [20]).**

Let *x* ∈ *ℝ* ^*n*^, *A* = *A* ^*T*^ ∈ *ℝ* ^*n*×*n*^, and *B* ∈ *ℝ* ^*m*×*n*^ such that rank{B}<n. The following statements are equivalent:

- *x* ^*T*^ *Ax* < 0, for all *Bx* = 0, *x* ≠ 0,
- *B* ^⊥^^*T*^ *AB* ^⊥^ < 0,
- ∃*X* ∈ *ℝ* ^*n*×*m*^: A+sym{XB}<0.

For convenient analysis, with the Kronecker product [21], the system (8) can be expressed as

(11) x˙t=IN⊗Axt+IN⊗BFIN⊗Cxt −σ−Γe⊗Inxt−ht, t∈tk,tk+1,

which imply

(12) x˙1t⋮x˙Nt︸x˙t=diagA,…,A︸IN⊗Ax1t⋮xNt︸xt +diagB,…,B︸IN⊗Bfy1t⋮fyNt︸Fyt −σ−γ11In−γ12In⋯−γ21In⋱⋱⋮⋱γNNIn︸Γe⊗In ×x1t−ht⋮xNt−hty1t⋮yNt︸yt=diagC,…,C︸IN⊗Cx1t⋮xNt,

where $\overset{-}{\sigma }=\sigma /\sum_{j=1,j\neq i}^{N}\gamma_{ij}$ and Γe=γ11-γ12⋯-γ21⋱⋱⋮⋱γNN with *γ*_*ii*_ = ∑_*j*≠*i*_ *γ*_*ij*_.

**Remark 5 .**

With the Kronecker product, the transformation from (8) to (11) has two advantages in the consensus analysis for the system (8): the first is the ease of mathematical representation, and the second is, in construction of the Lyapunov-Krasovskii functional, the applicability of the relation between the use of the Kronecker product with the matrix *U* defined in Lemma 3 and the term ‖*x*_*i*_(*t*) − *x*_*j*_(*t*)‖ stated in the condition (9) (see the equality (10)). As a result, based on the Kronecker product and Lemma 3, the consensus problem of the system (8) is converted into the Lyapunov stability problem of the transformed system (11).

## 3. Main Results

For simplicity of matrix and vector notations in Theorem 6, the following scalars and matrices are defined as

(13) ν1t=1tk−t+hM∫t−hMtkxsds,ν2t=1t−tk∫tktxsds,ϖt=1t−tk∫tktx˙sds,ζt=colCxtxt,xtk,xt−hM,x˙t,ν1t,1111111111ν2t,ϖt,fCxt,Υ=IN⊗A−σ−Γe⊗In0−IN⊗In000IN⊗Bxijt=xit−xjt,fCxijt=fCxit−fCxjt,ζijt=colCxijtxijt,xijtk,xijt−hM,x˙ijt,11111111111ν1ijt,ν2ijt,ϖijt,fCxijt,Υij=Aσ−γijNIn0−In000B,Π1,1ht=e1tk−t+hM︸hM−hte5t−tk︸hte6,Π1,2=e4−e3e1,Ξ1ht=symΠ1,1htPΠ1,2T+e1Qe2TΞ1ht=−e3Q3T+hM2e4Re4T−e2T−e3Te2T+e3T−2e5Te1T−e2Te1T+e2T−2e6TTΞ1ht=×diagR,3RM⋆diagR,3R︸ΩΞ1ht=×e2T−e3Te2T+e3T−2e5Te1T−e2Te1T+e2T−2e6T,Ξ2ht=tk−t+hM︸hM−hte4Se4T−t−tk︸hte7Se7T,Ξ3=−syme8−e1CTL−De8−e1CTL+T,Ξht=Ξ1ht+Ξ2ht+Ξ3,

where *e*_*i*_ ∈ *ℝ* ^(7*n*+*n*_*y*_)×*n*^ (*i* = 1,2,…, 8) are the block entry matrices; for example, *e*_2_^*T*^ *ζ*_*ij*_(*k*) = *x*_*ij*_(*t*_*k*_) and *e*_8_^*T*^ *ζ*_*ij*_(*t*) = *f*(*Cx*_*ij*_(*t*)).

**Theorem 6 .**

For a given positive scalar *h*_*M*_, the node in the system (8) is consented, if there exist matrices *𝒫* = [*P*_*ij*_] ∈ *𝕊*_+_^3*n*^, *Q* ∈ *𝕊*_+_^*n*^, *R* ∈ *𝕊*_+_^*n*^, *S* ∈ *𝕊*_+_^*n*^, *ℳ* = [*M*_*ij*_] ∈ *ℝ* ^2*n*×2*n*^, and diagonal matrix *D* ∈ *𝕊*_+_^*n*_*y*_^ satisfying the following LMIs for 1 ≤ *i* < *j* ≤ *N*:

(14) $$\begin{array}{c} {\left[\left(j-i\right)\Upsilon_{ij}^{⊥}\right]}^{T}\Xi_{k}\left[\left(j-i\right)\Upsilon_{ij}^{⊥}\right]<0\;\left(k=1,2\right), \end{array}$$

(15) $$\begin{array}{c} \Omega >0, \end{array}$$

where Ξ_*i*_ is the two vertices of Ξ_[*h*(*t*)]_ with the bounds of *h*(*t*), that is, 0 if *k* = 1 and *h*_*M*_ if *k* = 2.

**Proof**

Define a matrix *U* as *U* = [*u*_*ij*_]_*N*×*N*_ with *u*_*ij*_ = *N* − 1 if *i* = *j*, and otherwise, *u*_*ij*_ = −1. Then, consider the Lyapunov-Krasovskii functional candidate given by

(16) $$\begin{array}{c} V=V_{1}+V_{2}, \end{array}$$

where

(17) V1=xt∫t−hMtkxsds∫tktxsdsTU⊗P11U⊗P12U⊗P13⋆U⊗P22U⊗P23⋆⋆U⊗P33 ×xt∫t−hMtkxsds∫tktxsds+∫t−hMtxTsU⊗Qxsds +hM∫t−hMt∫stx˙TuU⊗Rx˙udu ds,V2=tk+1−t∫tktx˙TsU⊗Sx˙sds.

Time-differentiating *V*_1_ leads to

(18) V˙1=2xt∫t−hMtkxsds∫tktxsdsTU⊗P11U⊗P12U⊗P13⋆U⊗P22U⊗P23⋆⋆U⊗P33 ×x˙t−xt−hMxt+xTtU⊗Qxt −xTt−hMU⊗Qxt−hM +hM2x˙TtU⊗Rx˙t −hM∫tktx˙TsU⊗Rx˙sds.

By Wirtinger-based inequality [22] and reciprocally convex approach [23], the integral term is bounded as

(19) −hM∫t−hMtx˙TsU⊗Rx˙sds =−hM∫t−hMtkx˙TsU⊗Rx˙sds  −hM∫tktx˙TsU⊗Rx˙sds ≤−hMtk−t+hM  ×ϕ1,1TtU⊗Rϕ1,1t+ϕ1,2TtU⊗3Rϕ1,2t  −hMt−tk  ×ϕ2,1TtU⊗Rϕ2,1t+ϕ2,2TtU⊗3Rϕ2,2t ≤−ϕ1,1tϕ1,2tT  ×diagU⊗R,U⊗3RU⊗M11U⊗M12U⊗M21U⊗M22⋆diagU⊗R,U⊗3R  ×ϕ1,1tϕ1,2t,

where *ϕ*_1,1_(*t*) = *x*(*t*_*k*_) − *x*(*t* − *h*_*M*_), *ϕ*_1,2_(*t*) = *x*(*t*_*k*_) + *x*(*t* − *h*_*M*_)−(2/(*t*_*k*_ − *t* + *h*_*M*_))∫_*t*−*h*_*M*__^*t*_*k*_^ *x*(*s*)*ds*, *ϕ*_2,1_(*t*) = *x*(*t*) − *x*(*t*_*k*_), and *ϕ*_2,2_(*t*) = *x*(*t*) + *x*(*t*_*k*_)−(2/(*t* − *t*_*k*_))∫_*t*_*k*__^*t*^ *x*(*s*)*ds*.

From Lemma 3, ${\dot{V}}_{1}$ can be bounded as

(20) $$\begin{array}{c} {\dot{V}}_{1}\leq \underset{1\leq i<j\leq N}{\sum }\zeta_{ij}^{T}\left(t\right)\Xi_{1\left[h\left(t\right)\right]}\zeta_{ij}\left(t\right). \end{array}$$

By Jensen inequality [24] and Lemma 3, an upper bound of ${\dot{V}}_{2}$ is obtained as

(21) V˙2=tk+1−tx˙TtU⊗Sx˙t −∫tktx˙TsU⊗Sx˙sds≤tk+1−tx˙TtU⊗Sx˙t −1t−tk∫tktx˙sdsTU⊗S∫tktx˙sds=tk+1−tx˙TtU⊗Sx˙t −t−tkϖTtU⊗Sϖt=∑1≤i<j≤NζijTtΞ2htζijt.

In addition, the following inequality holds for any positive diagonal matrix *D*:

(22) 0≤−2fIN⊗Cxt−IN⊗L−IN⊗CxtT ×U⊗DfIN⊗Cxt−IN⊗L+IN⊗Cxt=−2fIN⊗Cxt−IN⊗L−CxtT ×U⊗DfIN⊗Cxt−IN⊗L+Cxt=∑1≤i<j≤NζijTt1111111×sym−e8−e1CTL−De8−e1CTL+T︸Ξ3ζijt.

Therefore, from (20) to (22), an upper bound of ${\dot{V}}_{\;}$ is

(23) $$\begin{array}{c} \dot{V}\leq \underset{1\leq i<j\leq N}{\sum }\zeta_{ij}^{T}\left(t\right)\underset{\Xi_{\left[h\left(t\right)\right]}}{\underset{︸}{\left(\Xi_{1\left[h\left(t\right)\right]}+\Xi_{2\left[h\left(t\right)\right]}+\Xi_{3}\right)}}\zeta_{ij}\left(t\right). \end{array}$$

Then, for *h*(*t*) → 0 and *h*(*t*) → *h*_*M*_, the following conditions hold

(24) ∑1≤i<j≤NζijTtΞhtζijt<0 ⟺α∑1≤i<j≤NζijTtΞ0ζijt+1−α  11×∑1≤i<j≤NζijTtΞhMζijt<0,

where *α* = (*h*_*M*_ − *h*(*t*))/*h*_*M*_.

Applying (i) and (iii) of Lemma 4 with the following equality:

(25) $$\begin{array}{c} \underset{1\leq i<j\leq N}{\sum }\left(j-i\right)\Upsilon_{ij}\zeta_{ij}\left(t\right)=0 \end{array}$$

leads to the following two conditions:

(26) $$\begin{array}{c} \underset{1\leq i<j\leq N}{\sum }\zeta_{ij}^{T}\left(t\right)\left(\Xi_{\left[0\right]}+sym\left\{X\Upsilon_{ij}\right\}\right)\zeta_{ij}\left(t\right)<0,\\ \underset{1\leq i<j\leq N}{\sum }\zeta_{ij}^{T}\left(t\right)\left(\Xi_{\left[h_{M}\right]}+sym\left\{X\Upsilon_{ij}\right\}\right)\zeta_{ij}\left(t\right)<0. \end{array}$$

Here, if the inequality $\Xi_{\left[h(t)\right]}+sym\{X\Upsilon_{ij}\}<0$ holds, then there exist positive scalars *ε*_1_ and *ε*_2_ such that

(27) $$\begin{array}{c} \Xi_{\left[0\right]}+sym\left\{X\Upsilon_{ij}\right\}<-\varepsilon_{1}I_{8n},\\ \Xi_{\left[h_{M}\right]}+sym\left\{X\Upsilon_{ij}\right\}<-\varepsilon_{2}I_{8n}. \end{array}$$

From (25), (26), and (27), we have

(28) V˙≤∑1≤i<j≤NζijTtΞht+symXΥijζijt<∑1≤i<j≤NζijTt−min⁡ε1,ε2I8nζijt<∑1≤i<j≤NxijTt−min⁡ε1,ε2Inxijt=∑1≤i<j≤N−min⁡ε1,ε2xijt2=∑1≤i<j≤N−min⁡ε1,ε2xit−xjt2.

By Lyapunov theorem and the definition for consensus (9), it can be guaranteed that the nodes in the nonlinear complex systems (8) are asymptotically consented.

In addition to this, in order to illustrate the process of obtaining (25), let us define

(29) $$\begin{array}{c} \Lambda =\left[\Lambda_{1},\Lambda_{2},\ldots ,\Lambda_{N}\right]=\left[N,N-1,\ldots ,1\right]⊗I_{n}\in R^{n\times nN}, \end{array}$$

where Λ_*k*_ ∈ *ℝ* ^*n*×*n*^ (*k* = 1,…, *N*).

Then, according to the proof of Theorem  1 in [9], we have the following zero equality:

(30) 0=ΛU⊗InΥζt=ΛU⊗InIN⊗A−σ−Γe⊗In0−IN⊗In000IN⊗Bζt=ΛU⊗A−σ−UΓe⊗In0−U⊗In000U⊗Bζt=ΛU⊗Axt−σ−ΛUΓe⊗Inxtk −ΛU⊗Inx˙t+ΛU⊗BfCxt.

By Lemma 3, the first term of (30) can be obtained as

(31) ΛU⊗Axt =NIn,N−1In,…,In︸n×nNU⊗A︸nN×nN  ×x1t,…,xNtT︸nN×1 =−∑1≤i<j≤NuijΛi−ΛjAxit−xjt =∑1≤i<j≤NΛi−ΛjAxit−xjt =∑1≤i<j≤NN+1−iIn11111111111−N+1−jInAxit−xjt =∑1≤i<j≤Nj−iAxit−xjt.

Similarly, the other terms of (30) are calculated as

(32) −σ−ΛUΓe⊗Inxtk =∑1≤i<j≤Nj−iσ−NγijInxit−ht−xjt−ht,−ΛU⊗Inx˙t =−∑1≤i<j≤Nj−iInx˙it−x˙jt,ΛU⊗BfCxt =∑1≤i<j≤Nj−iBfCxit−fCxjt.

Then, (30) can be rewritten as

(33) $$\begin{array}{c} 0=\Lambda \left(U⊗I_{n}\right)\Upsilon \zeta \left(t\right)=\underset{1\leq i<j\leq N}{\sum }\left(j-i\right)\Upsilon_{ij}\zeta_{ij}\left(t\right). \end{array}$$

Finally, reapplying (ii) and (iii) of Lemma 4 to (26), the following inequalities can be obtained

(34) $$\begin{array}{c} \underset{1\leq i<j\leq N}{\sum }{\left[\left(j-i\right)\Upsilon_{ij}^{⊥}\right]}^{T}\Xi_{\left[0\right]}\left[\left(j-i\right)\Upsilon_{ij}^{⊥}\right]<0,\\ \underset{1\leq i<j\leq N}{\sum }{\left[\left(j-i\right)\Upsilon_{ij}^{⊥}\right]}^{T}\Xi_{\left[h_{M}\right]}\left[\left(j-i\right)\Upsilon_{ij}^{⊥}\right]<0. \end{array}$$

From (34), if the LMIs (14) satisfy, then the condition (24) subject to (25) holds. This completes our proof.

For comparison, the following corollary is introduced.

**Corollary 7 .**

For a given positive scalar *h*_*M*_, the node in the system (1) under the procotol (5) with time-varying sampled data is consented, if there exist matrices *𝒫* = [*P*_*ij*_] ∈ *𝕊*_+_^3*n*^, *Q* ∈ *𝕊*_+_^*n*^, *R* ∈ *𝕊*_+_^*n*^, *S* ∈ *𝕊*_+_^*n*^, *ℳ* = [*M*_*ij*_] ∈ *ℝ* ^2*n*×2*n*^, and diagonal matrix *D* ∈ *𝕊*_+_^*n*_*y*_^ satisfying the following LMIs for 1 ≤ *i* < *j* ≤ *N*:

(35) $$\begin{array}{c} {\left[\left(j-i\right){\tilde{\Upsilon }}_{ij}^{⊥}\right]}^{T}\Xi_{k}\left[\left(j-i\right){\tilde{\Upsilon }}_{ij}^{⊥}\right]<0,\;\left(k=1,2\right), \end{array}$$

(36) $$\begin{array}{c} \Omega >0. \end{array}$$

**Proof**

Replacing *Υ*_*ij*_ with ${\tilde{\Upsilon }}_{ij}=\left[\begin{array}{cccccccc} A & \sigma d_{ij}NI_{n} & 0 & -I_{n} & 0 & 0 & 0 & B \end{array}\right]$ in the proof of Theorem 6 leads to (35).

## 4. Numerical Example

In this section, one numerical example will be presented to illustrate the effectiveness of the proposed criteria in this paper.

**Figure 3:**
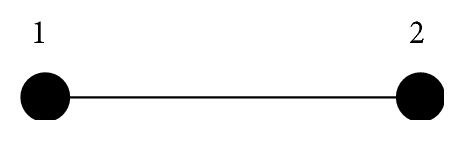
2-node information flow.

Consider 2-node information flow drawn in Figure 3 consisted of the Chua's circuit [25] given by

(37) x˙i1t=αxi2t−hxi1t,x˙i2t=xi1t−xi2t+xi3t,x˙i3t=−βxi2t, i=1,2

with the nonlinear function *h*(*x*_*i*1_(*t*)) = *m*_1_ *x*_*i*1_(*t*)+(1/2)(*m*_0_ − *m*_1_)(|*x*_*i*1_(*t*) + *c* | −|*x*_*i*1_(*t*) − *c*|), where parameters *m*_0_ = −1/7, *m*_1_ = 2/7, *α* = 9, *β* = 14.28, and *c* = 1 and its Lur's form can be rewritten with

(38) $$\begin{array}{c} A=\left[\begin{array}{ccc} -\alpha m_{1} & \alpha & 0\\ 1 & -1 & 1\\ 0 & -\beta & 0 \end{array}\right],\;\;B=\left[\begin{array}{c} -\alpha \left(m_{0}-m_{1}\right)\\ 0\\ 0 \end{array}\right],\\ C^{T}=\left[\begin{array}{c} 1\\ 0\\ 0 \end{array}\right]. \end{array}$$

For the above system, the maximum interval of *t*_*k*+1_ − *t*_*k*_ (=*h*_*M*_) for fixed coupling strength *σ* = 1 is compared between degree and edge betweenness centralities as shown in Table 1. From Table 1, it can be seen that the result with the edge betweenness centrality measure for this example gives larger maximum interval of *t*_*k*+1_ − *t*_*k*_ (=*h*_*M*_) than the one with the degree centrality measure.

Moreover, the elements of matrix Γ_*e*_ can be calculated as

(39) γ12=∑k≠lgkle12gkl=g12e12g12+g21e12g21=11+11=2,γ21=∑k≠lgkle21gkl=g12e21g12+g21e21g21=11+11=2,γ11=∑j≠iγ1j=γ12=2,  γ22=∑j≠iγ2j=γ21=2.

**Figure 4:**
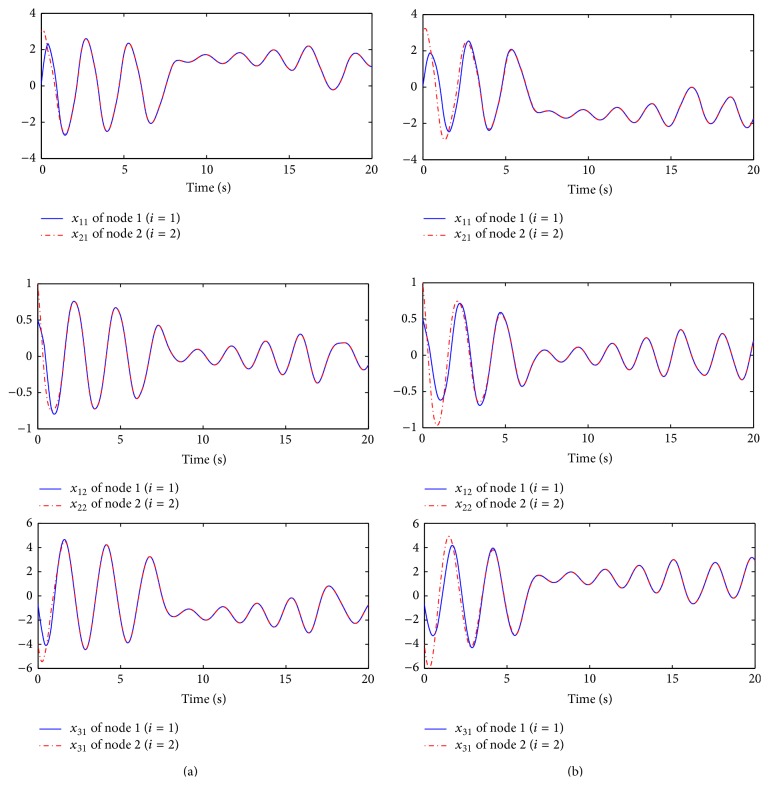
State trajectories of each node: (a) degree and (b) edge.

**Figure 5:**
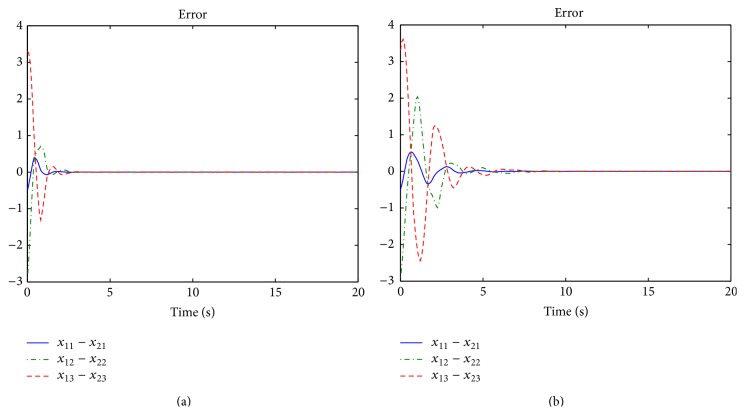
Error trajectories of each node: (a) degree and (b) edge.

**Figure 6:**
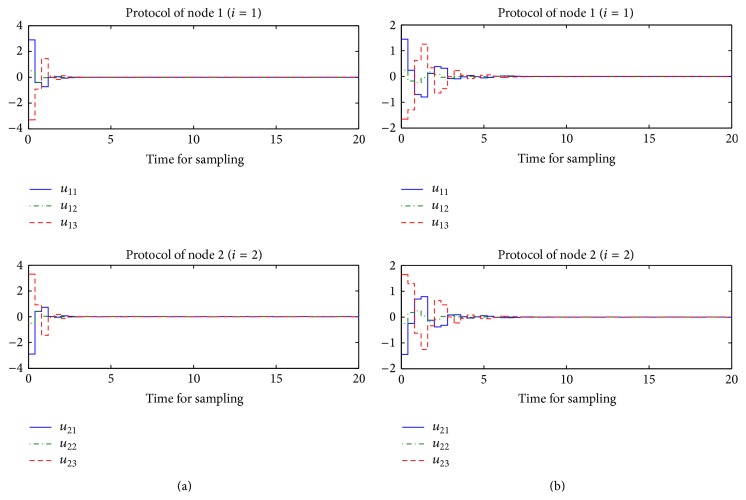
Protocol trajectories of each node: (a) degree and (b) edge.

**Figure 7:**
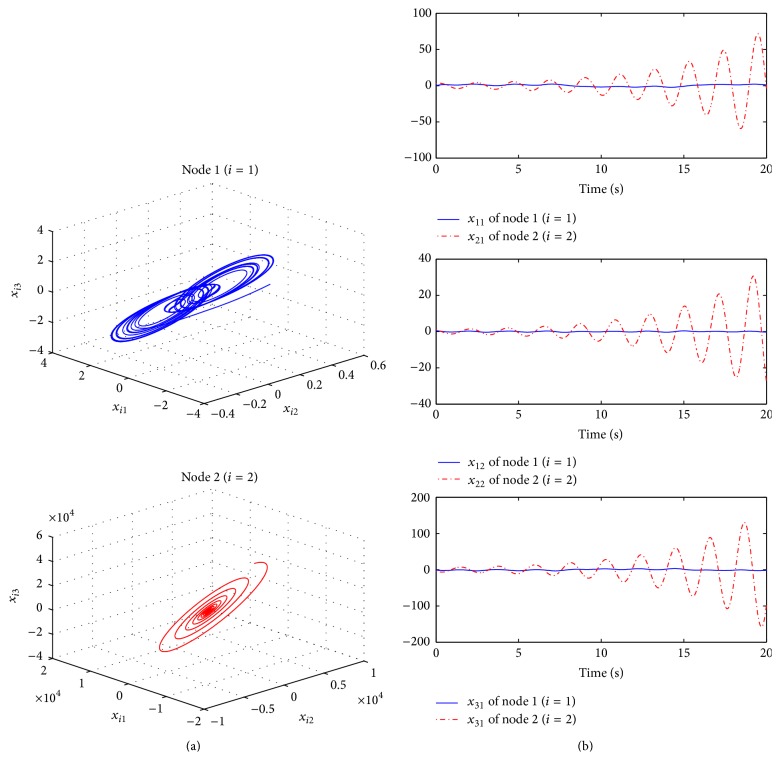
Results without the consensus protocol, that is, *u*_*i*_(*t*_*k*_) = 0: (a) phase and (b) each state.

However, the system performance with the edge betweenness centrality measure is more poor and needs more protocol input than the one with the degree centrality measure. For comparison between two measure cases, the sampling interval *t*_*k*+1_ − *t*_*k*_ (=*h*_*M*_) is assumed to be 0.4.  Figure 4 shows that the states with the responses consent to the same behavior under two measure cases for the given initial states of the nodes *x*_1_^*T*^(0) = [0.1  0.5–0.7] and *x*_2_^*T*^(0) = [3  1–4]. In Figure 5, their error trajectories are shown. Here, the case of the edge betweenness centrality measure indicates the poor performance. Thus, it can be confirmed that it is necessary to consider the global information for network structure as mentioned in Remark 1. Their corresponding protocol inputs can be identified in Figure 6. In addition to this, without the protocol, the behaviors of two nodes are different as shown in Figure 7.

## 5. Conclusions

In this paper, the consensus analysis for nonlinear complex systems under time-varying sampled-data protocol has been conducted. The information for network structure is measured by edge betweenness centrality, which has the global information while the degree centrality has the local one. To achieve this, by constructing the simple Lyapunov-Krasovskii functional, sufficient conditions for guaranteeing asymptotic consensus of such systems have been derived in terms of LMIs. One numerical example has been given to show the usefulness of the proposed model.

## Figures and Tables

**Table 1:** Comparison with fixed coupling strength σ = 1.

Measures	Methods	Structure	*t*_*k*+1_ − *t*_*k*_ (=*h*_*M*_)
Degree centrality	Corollary 7	${\;}^{*}\Gamma_{d}=\left[\begin{array}{cc} 1 & -1\\ -1 & 1 \end{array}\right]$	0.41

Edge betweenness centrality	Theorem 6	$\Gamma_{e}=\left[\begin{array}{cc} 2 & -2\\ -2 & 2 \end{array}\right]$	0.49

^*^is the Laplacian matrix of graph drawn in Figure 3.
